# A Personalized, Texting-Based Conversational Agent to Address Sleep Disturbance in Individuals Who Have Survived Breast Cancer: Protocol for a Pilot Waitlist Randomized Controlled Trial

**DOI:** 10.2196/62712

**Published:** 2025-07-14

**Authors:** Chi-shan Tsai, Warren Szewczyk, Michelle Drerup, Jason Liao, Alexi Vasbinder, Heather Greenlee, Jaimee L Heffner, Rachel Yung, Kerryn W Reding

**Affiliations:** 1 School of Nursing University of Washington Seattle, WA United States; 2 School of Public Health University of Washington Seattle, WA United States; 3 Neurological Insititute Cleveland Clinic Cleveland, OH United States; 4 Public Health Sciences Division Fred Hutchinson Cancer Center Seattle, WA United States; 5 Clinical Research Division Fred Hutchinson Cancer Center Seattle, WA United States; 6 School of Medicine University of Washington Seattle, WA United States

**Keywords:** breast cancer, conversational agent, sleep disturbance, randomized controlled trial, cognitive behavioral therapy for insomnia, physical activity

## Abstract

**Background:**

Sleep disturbance is one of the most common health concerns reported by individuals who have survived breast cancer (BC) and is associated with poor quality of life (QoL) and greater mortality after treatment. Cognitive behavioral therapy for insomnia (CBTi) has shown efficacy for improving sleep and QoL for this population. Considered the gold standard for insomnia treatment, CBTi can be delivered remotely, including via digital intervention. Despite the potential for wider dissemination of CBTi via digital means, these modalities have unique challenges, including technology barriers and poor adherence. We developed a conversational agent (CA) to deliver CBTi via a SMS text messaging intervention, supported by mobile-ready web content. Named “Cecebot,” this CA delivers sleep education, implements sleep compression, provides just-in-time interventions for sleep-disrupting behaviors, and includes enhanced support for physical activity (PA) beyond what is typically included in CBTi. This represents a novel modality for a CBTi and PA intervention among individuals who have survived BC.

**Objective:**

We aim to examine the safety and acceptability of the Cecebot intervention, developed by an academic partnership between Dr Reding’s research team and Moby Inc, for individuals who have survived BC and experience symptoms of insomnia, and to explore its efficacy.

**Methods:**

This trial will recruit 60 individuals who have survived BC and are experiencing moderate to severe sleep disturbance. Participants will be assigned to the Cecebot intervention or waitlist control group at a 1:1 ratio. The treatment group will receive the Cecebot intervention during weeks 1-6 of the study, while the waitlist control condition will receive the Cecebot intervention during weeks 6-12. The Cecebot intervention uses SMS text messaging technology paired with a Fitbit. Participants will be assessed at baseline, week 6, and week 12. Measurements will include feasibility and acceptability and will explore the effect of the Cecebot intervention. Feasibility will be assessed through recruitment, enrollment, and retention rates. Acceptability will be evaluated using a satisfaction survey and open-ended responses. Quantitative analysis, such as *t* test, Fisher exact tests, and generalized linear models, will be used to assess feasibility, baseline group differences, and the outcomes of the intervention.

**Results:**

Recruitment of participants began in Fall 2024. The completion of data collection is anticipated to be by Fall 2025.

**Conclusions:**

The study results will give insight into the potential for an SMS text messaging–based CA to improve sleep in individuals who have survived BC and experience sleep disturbances.

**Trial Registration:**

ClinicalTrials.gov NCT06392789; https://clinicaltrials.gov/study/NCT06392789

**International Registered Report Identifier (IRRID):**

DERR1-10.2196/62712

## Introduction

### Background

More than 4 million women with a history of breast cancer (BC), often termed individuals who have survived BC [1], are estimated to be living in the United States today [2]. Though improved BC treatments are contributing to increased survival rates, one-third of these individuals endure long-lasting side effects, such as fatigue and depression [3]. Sleep disturbance is one of the most common effects, with more than 50% of people who have survived BC experiencing sleep disturbances after treatment [4-6]. Lower sleep quality is associated with worse psychosocial outcomes, more severe symptoms of fatigue and pain, and a greater need for supportive care [4].

Cognitive behavioral therapy for insomnia (CBTi) is recommended as a first-line treatment for insomnia symptoms since it is safe and effective, exhibiting robust and persistent reduction in symptoms of insomnia and quality of life (QoL) improvements for individuals who have survived BC in well-designed clinical trials [7-9]. However, the need for treatment exceeds the availability of trained CBTi providers [10], and implementation at a large scale is relatively expensive. Patient burden is a critical concern, as poor sleep and fatigue may interact with practical and motivational barriers to limit engagement with standard CBTi, particularly after BC and treatment [11]. Digital health modalities may offer less burdensome and more scalable intervention opportunities, which may be particularly useful for people with barriers to traditional health care. However, intervening via remote telemedicine has limitations. Technological barriers (eg, incompatible hardware, software, connectivity, or scheduling) can prevent access to the intervention, and remote digital modalities generally entail a more self-directed experience than one with a live therapist or coach [12].

We developed a CBTi intervention delivered via SMS text messaging that is supported by mobile-ready web content. Named Cecebot, the 6-week intervention takes the form of a rule-based conversational agent (CA) and integrates passive data collection from Fitbit and active collection from participants. CAs offer the opportunity for more accessible and personalized digital health tools, which may promote participant retention and intervention effectiveness while remaining scalable and cost-efficient [13].

### Study Objectives

The objectives of this pilot randomized trial are (1) to examine the acceptability of the Cecebot intervention and the feasibility of the clinical trial for individuals who have survived BC with chronic moderate-to-severe insomnia and (2) to explore the efficacy of Cecebot for reducing insomnia symptoms, improving QoL, increasing physical activity (PA) frequency, reducing symptoms of depression, and decreasing fatigue.

## Methods

### Study Overview

**Figure 1:**
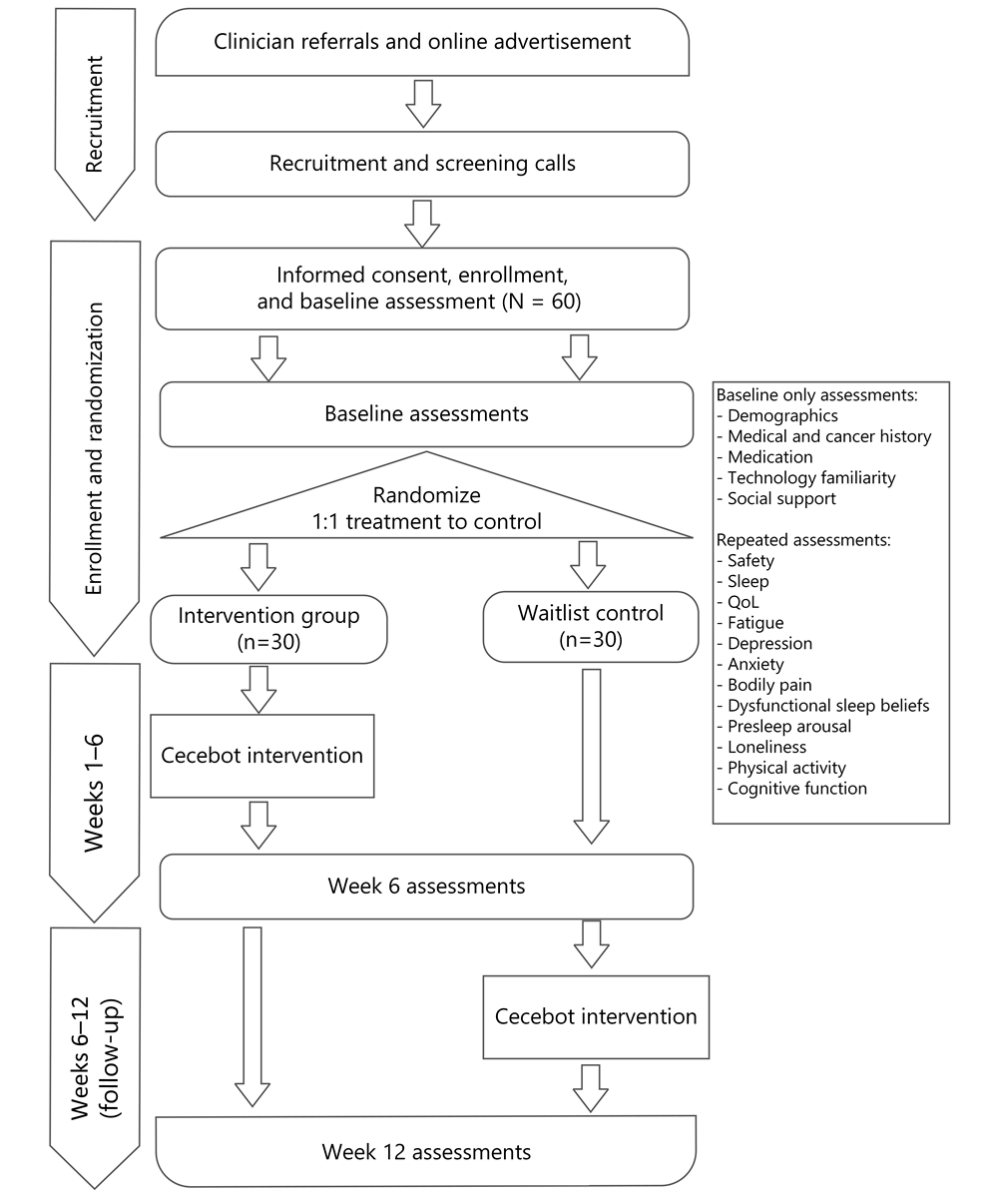
Flow diagram of Cecebot trial design. QoL: quality of life.

The study is a fully remote, 12-week, 2-arm pilot randomized controlled trial (RCT; N=60) for women with early-stage BC who have completed BC treatment and are experiencing moderate sleep disturbance (refer to Figure 1 for the study schema). Preliminary acceptability, feasibility, and efficacy of the 6-week Cecebot intervention will be assessed in comparison to a passive waitlist control group with 1:1 assignment of intervention to control.

All participants will receive the full intervention. The treatment condition will use Cecebot from weeks 1-6, and the waitlist control condition will receive the intervention during weeks 7-12. All participants will complete outcome measures remotely via a secure web form at baseline, 6 weeks, and 12 weeks. This protocol follows the CONSORT‐EHEALTH (Consolidated Standards of Reporting Trials of Electronic and Mobile Health Applications and Online Telehealth) (refer to Multimedia Appendix 1 for the checklist) and SPIRIT (Standard Protocol Items: Recommendations for Interventional Trials) guidelines (refer to Multimedia Appendix 2) [14,15].

### Study Population and Recruitment

This study will recruit 60 participants through a multipronged approach. The primary methods will be via provider referral from oncology clinics, recontact of previous study participants in unrelated BC research projects, medical record invitation, and web-based advertising. Candidate participants will initially complete a secure web-based screening survey to assess basic eligibility criteria. Those who are eligible after the first pass will be asked further screening questions by phone with trained staff to assess more sensitive eligibility, such as psychiatric comorbidities. All eligibility criteria will be self-reported, with some assessed via validated clinical assessment (eg, current insomnia) and others with self-report of past or current diagnosis (eg, noninsomnia sleep disorder). The inclusion and exclusion criteria are listed in Textbox 1.

**Inclusion and exclusion criteria.**

**Inclusion criteria**

- Age ≥18 years.
- Diagnosis of stage I-III invasive breast cancer in the previous 5 years.
- Female gender.
- Clinically significant insomnia symptoms, defined as a T-score ≥55 on the Patient-Reported Outcomes Measurement Information System (PROMIS) Sleep Disturbance 8a.
- Self-report of insomnia symptoms lasting ≥3 months.
- Eastern Cooperative Oncology Group (ECOG) performance status of 0-2, indicating the ability to perform activities of daily living.
- Owns a smartphone with internet connectivity.
- Proficiency with written and spoken English.
- Willing and able to complete the intervention with their smartphone.

**Exclusion criteria**

- Self-report of a prior diagnosis of restless leg syndrome, periodic leg movement disorder, narcolepsy, or rapid eye movement behavior disorder.
- Current high risk for sleep apnea, assessed with modified STOP (Snoring, Tiredness, Observed apnea, and high blood Pressure) criteria.
- Current shiftwork or regular environmental disturbance (eg, infant child).
- Actively receiving chemotherapy or radiation (endocrine therapy permitted) or plans to receive chemotherapy, radiation, or surgery in the next 6 months.
- Contraindications to cognitive behavior therapy for insomnia include self-report of active psychosis, uncontrolled bipolar disorder, active suicidality, active substance use disorder (moderate or greater severity, as identified by the Tobacco, Alcohol, Prescription medication, and other Substance), or severe depression defined as the 8-item Patient Health Questionnaire (PHQ-8) depression scale ≥20.
- Use of prescribed sleep medication >3 times per week.
- Previously received cognitive behavior therapy for insomnia with a professional therapist.
- Previously participated in user testing of Cecebot.
- Unwilling or unable to complete study procedures.

Because Cecebot is a self-directed, text-based digital intervention, we expect that both reading skills and resource access are important facilitating factors for benefiting from the intervention. These factors are influenced, at least in part, by education, but most digital health versions of CBTi have been tested in highly educated populations. To investigate the feasibility and acceptability for people with less education, we aim to enroll a sufficient proportion of participants (50%) who do not have a bachelor’s degree.

All eligible participants will provide informed consent before the collection of baseline data. Following baseline data collection, participants will be randomly assigned to the intervention or the waitlist control group at a 1:1 ratio. Permuted block randomization will be used, stratified by whether participants are older than 55 years and whether they have a bachelor’s degree. The random allocation procedure will be conducted using the randomization module in Research Electronic Data Capture (REDCap), developed by Paul A Harris at Vanderbilt University, by qualified and trained research staff.

### Intervention Overview

The 6-week Cecebot intervention is based on the core content of CBTi and uses Go! To Sleep, a 6-week self-directed web version of CBTi [16] previously developed by Dr Michelle Drerup that has enrolled over 1500 patients with insomnia. Cecebot was developed with an iterative design process that involved recruiting individuals who have survived BC and conducting multiple rounds of interviews and user testing to tailor the intervention for this population. Preferences for format, features, and content were solicited in focus groups (n=13, 3 groups) and individual interviews (n=3) to inform the initial design, after which a 1-week prototype version was developed and subsequently tested by individuals who have survived BC to elicit feedback. This feedback was synthesized and used to develop the full 6-week intervention.

**Figure 2:**
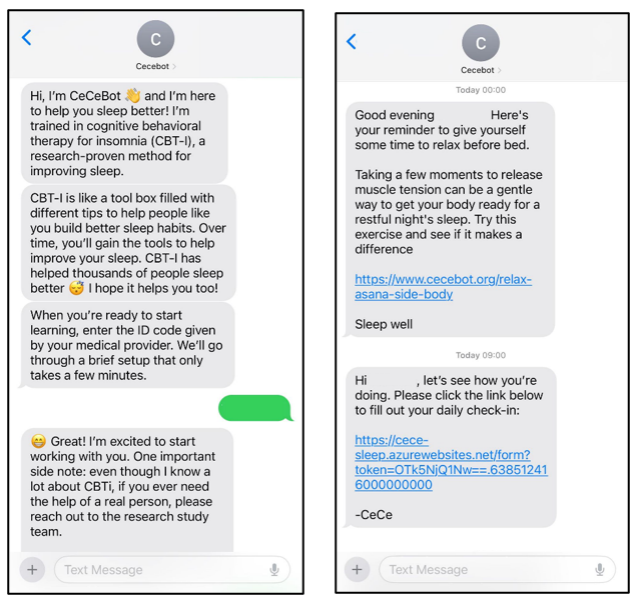
Example message of Cecebot.

Cecebot interacts with participants via SMS and is supported by mobile-ready web content. There are four core components: (1) tailored CBTi education delivered through SMS conversations and supporting web content (refer to Figure 2), (2) implementation of a sleep compression protocol based on web-based daily sleep diaries, (3) passive PA tracking via a consumer-grade activity tracker worn 24/7 during the intervention, and (4) accountability for sleep and PA behavior change using individually tailored messages. Cecebot is written in English, and the language is designed to be conversational, uncomplicated (approximately seventh-grade reading level), and empathic to facilitate accessibility by a broad audience and to minimize the cognitive burden of engaging in the intervention.

**Figure 3:**
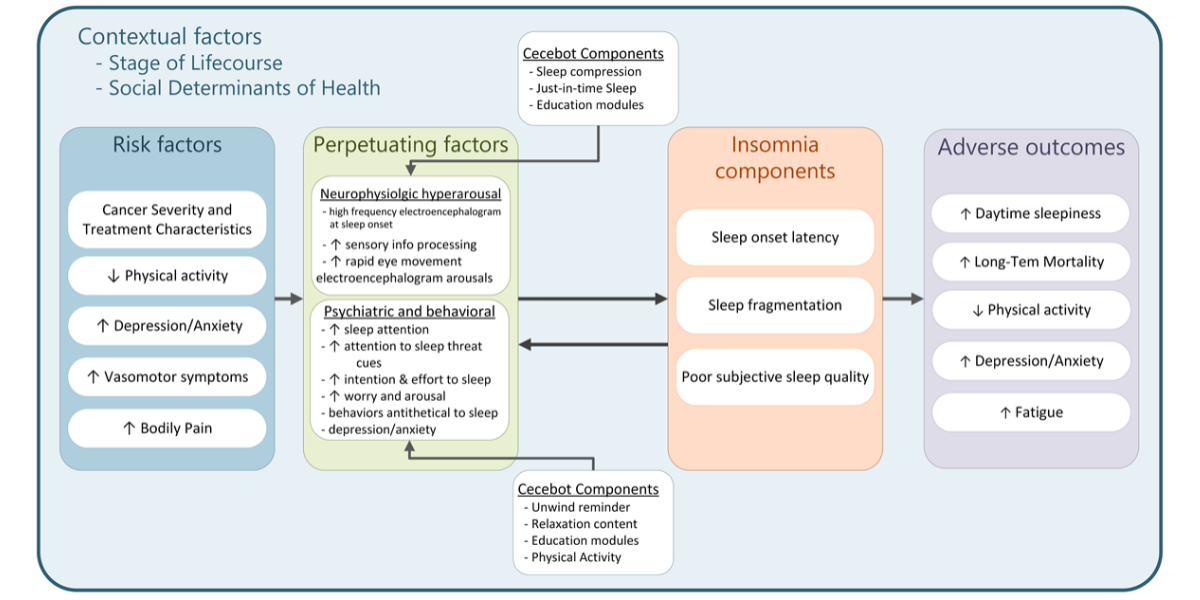
Conceptual model of insomnia in breast cancer and the intervention effects of Cecebot (adapted from Levenson et al [17]).

The education component focuses on core CBTi concepts (eg, the 3P model, stimulus control, sleep hygiene, sleep-related cognitions, and relaxation) delivered via SMS modules 2-4 times a week. Each educational component is designed to be completed in approximately 10 minutes (Table 1 and Figure 3 [17]). In total, there are 20 modules delivered over 6 weeks (42 days). These modules are pseudo-conversations, where Cecebot presents information in a conversational tone and often asks the participant a reflective question, which can be answered with a free response. Cecebot gives the same response regardless of participant input. Web content is provided as a link within most conversations and offers additional details for the concepts presented via text. All supporting web content is designed to be accessed on a mobile device. Over 25% of the education modules that present new information have a tailored component. These modules provide additional content based on participant answers provided during an “intake” conversation completed with Cecebot at the beginning of the intervention. For example, participants are asked about common dysfunctional sleep beliefs, such as “When I feel tired, have no energy, or just seem not to function well during the day, it is because I did not sleep well the night before.” Endorsing a specific dysfunctional belief tailors a later education conversation to specifically address that belief. Sleep compression, a less intensive version of the sleep restriction therapy that is part of CBTi, is algorithmically implemented in once-per-week SMS conversations based on sleep efficiency and sleep need over the past week, which are calculated from sleep diaries submitted daily by each participant [18]. Cecebot tracks items on the 4-item Sleep Need Questionnaire over each 7 days so that sleep needs can be used for the algorithm [19,20]. If a participant’s sleep efficiency is less than 85%, Cecebot recommends reducing time in bed by 30 minutes. If a participant’s sleep efficiency is greater than 85% but they have high sleep needs, Cecebot suggests increasing time in bed by 30 minutes. Sleep compression is considered low risk with minimal side effects, making it a better fit for an unsupervised digital intervention than sleep restriction.

**Table 1:** Cecebot education module schedule and conceptual areas addressed.

Conceptual area and module title			Timing of the module within the intervention					
			Week		Day			
**Insomnia basics** **in the context of** **cancer**								
	“The Insomnia Cycle” (3P model)^a^	1		1				
	Sleep drive	1		2				
	Stimulus control	1		3				
	Sleep compression	1		6				
**Sleep** **hygiene and physical activity**								
	Take a time-out	2		2				
	Sleep and physical activity	2		3				
	Sleep or wake clock	2		5				
	Sleep hygiene	2		7				
**Review**								
	Review week 1	3		2				
**Managing barriers**								
	Managing sleep barriers^a^	3		4				
	Physical activity barriers^a^	3		6				
	Cancer-related barriers^a^	4		2				
**Addressing anxiety and restructuring cognitive**								
	Helpful worry time	4		3				
	Changing thought patterns^a^	4		5				
	Gratitude and sleep	4		7				
	Physical activity and sleep	5		2				
**Review**								
	Review week 1	5		5				
	Review week 3 and 4 content	6		2				
**A postintervention action plan**								
	Physical activity action plan	6		4				
	Sleep action plan	6		7				

^a^Indicates a tailored module.

In addition to 4 tailored sleep modules, one tailored module addresses barriers to PA experienced by the participant. This module supports the PA content and motivation offered in Cecebot, which is augmented beyond what is typically provided in standard CBTi. Though a causal link between increased PA and improved sleep is not firmly established, interventional evidence from individuals who have survived BC suggests PA interventions can reinforce improved sleep quality [21]. This evidence, along with the benefits of PA on fatigue and fitness for this population, justifies increased attention on PA within the intervention.

The enhanced PA focus of Cecebot is supported by integration with a Fitbit consumer activity tracker (Fitbit Inc). Cecebot uses the Fitbit application programming interface to collect participant step count, heart rate, and sleep data regularly. Participants can request a summary of their previous week’s step count at any time and receive a summary at each weekly check-in. Passive activity tracking also supports Cecebot’s just-in-time (JIT) functionality. JIT behavior interventions aim to provide microinterventions with temporal relevance to the behavior being changed or initiated [22]. Cecebot checks PA levels every 4 days and sends unique messages based on the mean 4-day step count observed—a low step count (<2000/d) prompts a message suggesting initiation of brief activity, while a high step count (>8000/d) prompts congratulations, and a middle-range step count (>6000/d) prompts a message nudging the participant toward increased PA. The PA JIT messages pair with weekly PA feedback and with PA education. The Cecebot website, maintained by the study, contains videos of workout routines tailored for individuals who have survived BC, led by a trained instructor.

Cecebot also provides sleep-specific JIT messages on a 4-day cycle. Participants endorse sleep-related behaviors from the previous day as part of each web-based daily sleep diary. The behaviors that can be tracked include sleep-promoting behaviors such as relaxation or a prebed routine, as well as sleep-disrupting behaviors such as late afternoon caffeine or nighttime alcohol. If a participant endorses a sleep-disrupting behavior on 2 or more days in 4 days, Cecebot sends a message aimed at intervening on the behavior. If more than one behavior is endorsed 2 or more times in the 4 days, the intervening message is tailored to the separate behaviors endorsed. These messages are drawn randomly from individual pools of messages that correspond to the behavior targeted for intervention (eg, a pool of caffeine messages).

Cecebot includes an optional prebed relaxation feature. If enabled, Cecebot sends a message 1 hour before the participant’s established bedtime to prompt them to begin unwinding and preparing for bed. Each message includes a web link to relaxation content created specifically for the intervention. The content is in audio format, based on feedback garnered from individuals who have survived BC during early rounds of design, which highlighted the potentially sleep-disrupting impact of video before bed. There are over 25 recordings that focus on either mindful meditation, breathing, progressive muscle relaxation, or light stretching.

### Measures

#### Baseline Measures

Following informed consent, enrolled participants will remotely complete surveys via REDCap, a secure data collection portal. Surveys completed only at baseline will collect basic demographics, medical and cancer history, current perceived social support, and familiarity with technology (Table 2). Perceived social support will be assessed with the 19-item Medical Outcomes Study Social Support Survey (MOS-SSS) questionnaire [23]. Technology familiarity will be measured using a short questionnaire developed by the research team. In addition to these assessments, the measures described below will be evaluated at baseline.

**Table 2:** Schedule of baseline and outcome measurements.

Outcome			Measure		Week 0 (baseline)		Week 6		Week 12
**Baseline only measures**									
	Demographics	Survey		✓					
	Medical history	Survey		✓					
	Technology literacy			✓					
	Social support	MOS-SSS^a^		✓					
**Outcome measures**									
	Feasibility	Multiple				✓		✓	
	Acceptability	Multiple				✓		✓	
	Safety	Interview				✓		✓	
	Insomnia	ISI^b^		✓		✓		✓	
	Sleep beliefs	DBAS-16^c^		✓		✓		✓	
	Presleep arousal	PSAS^d^		✓		✓		✓	
	Quality of life	SF-36^e^		✓		✓		✓	
	Fatigue	FACIT-F^f^		✓		✓		✓	
	Depression	PHQ-8^g^		✓		✓		✓	
	Anxiety	GAD-7^h^		✓		✓		✓	
	Bodily pain	PROMIS Interference^i^		✓		✓		✓	
	Bodily pain	PROMIS Intensity^j^		✓		✓		✓	
	Cognitive function	FACT-Cog^k^		✓		✓		✓	
	Physical activity	IPAQ-SF^l^		✓		✓		✓	

^a^MOS-SSS: Medical Outcomes Study Social Support Survey.

^b^ISI: insomnia severity index.

^c^DBAS-16: 16-item Dysfunctional Beliefs and Attitudes About Sleep Scale.

^d^PSAS: Pre-sleep Arousal Survey.

^e^SF-36: 36-item Short Form Survey.

^f^FACIT-F: Functional Assessment of Chronic Illness Therapy-Fatigue.

^g^PHQ-8: Patient Health Questionnaire-8.

^h^GAD-7: Generalized Anxiety Disorder 7-item.

^i^PROMIS-Interference: Patient-Reported Outcomes Measurement Information System Pain Interference.

^j^PROMIS-Intensity: Patient-Reported Outcomes Measurement Information System Pain Intensity.

^k^FACT-Cog: Functional Assessment of Cancer Therapy-Cognition Function.

^l^IPAQ-SF: International Physical Activity Questionnaire-Short Form.

#### Outcomes

Several hypothesized treatment effects of the intervention will be assessed at each time point to determine the effect of Cecebot on health outcomes. These include insomnia, QoL, PA, depression, anxiety, and cognitive function. In addition, putative treatment mechanisms will be assessed, including dysfunctional sleep cognitions, presleep arousal, and knowledge of CBTi concepts and principles.

Insomnia and sleep quality will be assessed with the insomnia severity index [24] and secondarily with daily web-based sleep diaries delivered through the intervention. The insomnia severity index (ISI) has 7 items (score range: 0-28), with higher scores reflecting more severe insomnia symptoms [25]. To measure treatment mechanisms that may partially mediate the effects of Cecebot, sleep cognitions will be assessed with the 16-item Dysfunctional Beliefs and Attitudes About Sleep Scale (DBAS-16) [26] presleep experience with the 16-item Pre-sleep Arousal Survey (PSAS) [27], and knowledge of CBTi concepts with a 13-item true or false questionnaire designed by the research team.

Quality of life will be assessed with the 36-item Short Form Survey (SF-36) [28], comprised of eight domains covering physical and mental health. Fatigue will be measured using the Functional Assessment of Chronic Illness Therapy-Fatigue (FACIT-F) [29], a 13-item survey with scores ranging from 0 to 52, where higher scores indicate greater functional impact of fatigue. Depression will be assessed with the PHQ-8 (8-item Patient Health Questionnaire) depression scale, and anxiety with the Generalized Anxiety Disorder 7-item (GAD-7) scale [30,31]. Higher scores indicate a higher level of depression or anxiety symptoms on these scales. The PROMIS (Patient-Reported Outcomes Measurement Information System)-Pain Interference [32] and PROMIS-Intensity will be used to measure the severity of pain and the influence of pain on their lives. Cognitive function will be measured using the 20-item perceived cognitive impairments subscale of the Functional Assessment of Cancer Therapy-Cognitive Function (FACT-Cog) questionnaire [33]. To assess the safety profile of Cecebot, adverse events will be collected at the 6- and 12-week timepoints with a brief structured interview administered by research staff. Participants will be instructed to report any severe adverse events to the research team during the interim between study visits.

Physical activity duration and intensity over the previous week will be assessed with the International Physical Activity Questionnaire-Short Form (IPAQ-SF) [34] survey. Wrist-worn consumer-grade actigraphy (Fitbit Charge 4, Fitbit Inc) will secondarily be used to assess PA by recording step count and movement pattern. The Fitbit measurement will collect daily reports from participants, and Fitbit metrics will be analyzed for quality control. Only days with greater than 7 hours of wear time will be considered valid.

To assess intervention acceptability and potential areas for improvement, participants will answer survey questions about their satisfaction with Cecebot with a survey developed by the research team, including 11 multiple-choice items and 4 open-ended questions. Since both the intervention and outcome measures are noninvasive, the risk of harm to participants is minimal. If a participant encounters discomfort and requests to withdraw, the research team will record their reason for withdrawal and conduct a safety assessment.

### Statistical Analysis

#### Primary Endpoint and Secondary Endpoints

The acceptability of the Cecebot intervention will be assessed in several ways. The daily sleep diaries will be used as a primary measure of adherence (the proportion of sleep diaries submitted) and treatment discontinuation, which will be defined as no sleep diary submission for 7 consecutive days or logging out of Cecebot. An all-cause discontinuation rate of 40% will be considered a threshold for acceptability. Treatment adherence will secondarily be assessed by comparing participant bedtimes and wake times to recommendations offered by Cecebot and by calculating (1) the proportions of weekly goal-setting conversations completed and (2) reflective prompts replied to within education modules. Qualitative satisfaction will be measured with a structured survey of participant opinions, including Likert scale assessments of satisfaction and opinions about Cecebot, along with open-ended questions allowing free text responses. Free text responses will be coded for themes. The adverse event rate will be another key indicator of acceptability. The number of participants experiencing an adverse event attributable to study procedures will be compared after the first 6 weeks to identify if any detectable differences are present between the treatment group and waitlist control group.

Trial feasibility will be assessed by calculating recruitment rates (proportion of participants enrolled of those approached), enrollment rate (proportion who enroll among those eligible), and loss to follow-up (proportion who do not complete the final study visit).

To determine if group differences are present after randomization, descriptive statistics of baseline characteristics (eg, age, cancer severity, and time since treatment) will be generated, and the treatment and control groups will be compared with *t* tests for continuous variables and chi-square tests for categorical variables. The Fisher exact test will be used for expected cell counts <5.

#### Exploratory Endpoints

We will conduct an exploratory analysis to investigate the effect of the Cecebot intervention on insomnia and other QoL outcomes. The analysis will analyze the following secondary outcomes: QoL, fatigue, depression, pain, and anxiety. Using generalized linear models to model difference scores, this study will examine the differences between intervention and waitlist control participants. The outcomes will be modeled as continuous variables using the measures described above. These analyses will be performed according to the intent-to-treat principle, where outcomes are analyzed by the intervention assigned, regardless of the level of adherence. The study will examine whether the intervention and control arms differ at baseline on pertinent variables using chi-square tests (for categorical variables) and *t* tests (for continuous variables). All analyses will use **α**=.05. A secondary per-protocol analysis accounting for adherence differences will be conducted. These results will be reported and interpreted along with the intent-to-treat analysis.

The study is powered to detect a crude effect size (Cohen f^2^) [35] of 0.16, which represents a moderate effect [36]. Controlling for baseline confounders (eg, age, race, cancer severity, endocrine or anti-human epidermal growth factor receptor 2 therapy, social support, and technology familiarity) increases the minimum detectable effect size to 0.30, a moderate-to-strong effect. Data analysis will be conducted using R version 4.5.1 (R Foundation for Statistical Computing). Missing data will be addressed using multiple imputations.

### Ethical Considerations

The protocol has been approved by the University of Washington institutional review board (19874) and is registered on ClinicalTrials.gov (NCT06392789). All participants are required to complete the informed consent form before participating in the study. The research team will ensure they are fully informed about the objectives and process of the study. All the collected data are securely stored in REDCap, and only authorized research team members have access to the data. Participants will receive a US $25 gift card for each completed study visit (baseline, 6 weeks, and 12 weeks), with a total possible reimbursement of US $75 in gift cards. Any significant modifications to the trial protocol will be documented in an amended protocol and submitted to the institutional review board for approval before implementation. Revised protocols will also be submitted to ClinicalTrials.gov. The research team will communicate with participants if applicable.

## Results

The final version of the protocol is version 6.0, dated January 31, 2025. Recruitment of participants to the pilot RCT commenced at the University of Washington and Fred Hutchinson Cancer Center in Fall 2024. As of February 25, 2025, this pilot RCT has recruited a total of 20 participants, with 11 participants in the intervention group and 9 participants in the waitlist control group. The completion of recruitment is planned to be completed by fall 2025. The research findings will be disseminated through peer-reviewed journal publications and presentations at academic conferences.

## Discussion

### Principal Findings

We hypothesize that the Cecebot intervention will improve sleep among individuals who have survived BC without causing any adverse effects, and we anticipate that the research process will be feasible and acceptable. We also expect to receive feedback on improvements and refinements from users. This study will provide useful information regarding the feasibility and acceptability of the Cecebot intervention for this population with sleep disturbance. The results will help define the potential value of an SMS-based CA for individuals who have survived BC. In addition, the data from the baseline, week 6, and week 12 follow-up timepoints will assist in exploring whether the intervention improves sleep quality in this population.

Previous studies have demonstrated that digital CBTi, such as mobile apps, may be beneficial in improving sleep [37,38]. Another randomized controlled trial using internet-delivered CBTi reported a reduction in the severity of insomnia and improved sleep quality among individuals who have survived BC [9]. However, limited studies are using an SMS-based CA in this population with insomnia. Furthermore, most studies of self-directed insomnia have included highly educated populations. Thus, evaluating the feasibility and effectiveness of this intervention, especially among those with less education, is a useful contribution to the area of sleep management during postcancer treatment survivorship. If the Cecebot intervention is proven to be feasible and effective in a future fully powered trial, it holds great potential for dissemination, which could lead to improved sleep quality and QoL for individuals who have survived BC.

### Strengths and Limitations

Cecebot is a tailored and SMS-based CA. Compared with traditional and face-to-face CBTi and PA interventions, it requires low resources and is inexpensive to execute. Therefore, this individualized intervention has the potential to be disseminated to a wider population, especially to marginalized groups. A limitation of the intervention is that, with its design as an SMS-based CA, the project cannot directly track how participants interact with Cecebot, as would be more feasible with an app-based solution. Instead, their behavior must be inferred from indicators such as which links were clicked and when. Another limitation is that Cecebot is a self-directed experience, which may be difficult to benefit from for participants with severe insomnia burden, a high burden of other cancer-related symptoms, or limited resource access.

### Conclusions

Cecebot is an intervention that overcomes resource-based barriers for marginalized groups via an SMS-based program that can be accessed at any time and place with minimal technological requirements and without the need to see a sleep specialist. This intervention has a high potential for future development, testing, and ultimately dissemination. The current study will evaluate the feasibility and acceptability of the intervention and explore the efficacy of an SMS-based conversational agent in individuals who have survived BC with sleep disturbances.

## Appendix

Multimedia Appendix 1 CONSORT-eHEALTH checklist (V 1.6.1).

Multimedia Appendix 2 SPIRIT (Standard Protocol Items: Recommendations for Interventional Trials) checklist.

## Abbreviations

BC: breast cancer
CA: conversational agent
CBTi: cognitive behavior therapy for insomnia
CONSORT‐EHEALTH: Consolidated Standards of Reporting Trials of Electronic and Mobile Health Applications and Online Telehealth
DBAS-16: 16-item Dysfunctional Beliefs and Attitudes About Sleep
FACIT-F: Functional Assessment of Chronic Illness Therapy-Fatigue
FACT-Cog: Functional Assessment of Cancer Therapy-Cognitive Function
GAD-7: Generalized Anxiety Disorder 7
IPAQ-SF: International Physical Activity Questionnaire-Short Form
ISI: insomnia severity index
JIT: just-in-time
MOS-SSS: Medical Outcomes Study Social Support Survey
PA: physical activity
PHQ-8: 8-item Patient Health Questionnaire
PROMIS: Patient-Reported Outcomes Measurement Information System
PSAS: Pre-sleep Arousal Survey
QoL: quality of life
RCT: randomized controlled trial
REDCap: Research Electronic Data Capture
SF-36: 36-item Short Form Health Survey
SPIRIT: Standard Protocol Items: Recommendations for Interventional Trials
